# Comparative Genomic and Transcriptomic Analysis Suggests the Evolutionary Dynamic of *GH3* Genes in Gramineae Crops

**DOI:** 10.3389/fpls.2019.01297

**Published:** 2019-10-15

**Authors:** Weilong Kong, Yue Zhang, Xiaoxiao Deng, Shuangmiao Li, Chenhao Zhang, Yangsheng Li

**Affiliations:** State Key Laboratory for Hybrid Rice, College of Life Sciences, Wuhan University, Wuhan, China

**Keywords:** *Glycoside hydrolase 3 (GH3)*, Gramineae crops, evolutionary patterns, evolutionary dynamic, gene duplication events

## Abstract

*Glycoside hydrolase 3* (*GH3*) gene family belongs to auxin-responsive gene families and is tightly linked with hormone homeostasis and signaling pathways. However, our knowledge about the evolutionary dynamic of *GH3* genes in Gramineae crops is limited. In this study, a comparative genomic and transcriptomic analysis was conducted to study evolutionary patterns and the driving selective forces of *GH3* gene family in six representative Gramineae crops, namely, *Setaria italica* (Si), *Zea mays* (Zm), *Sorghum bicolor* (Sb), *Hordeum vulgare* (Hv), *Brachypodium distachyon* (Bd), and *Oryza sativa* ssp. *japonica* (Os). A total of 17, 13, 11, 9, 8, and 11 GH3 proteins (GH3s) were identified in Si, Zm, Sb, Hv, Bd, and Os, respectively. Phylogenetic, conserved motif, and gene structural analyses could divide all GH3s into two groups (I and II), and all GH3s consisted of seven orthogroups (Ors) on the basis of Or identification result. We further found that genes in the same Or showed similar sequence and structural features, whereas genes in the same groups exhibited intrinsic differences in exon numbers and intron lengths. These results revealed *GH3* genes in the same groups have been differentiated. Obvious differences in total numbers of *GH3* genes, Ors, and duplication events among these six tested Gramineae crops reflected lineage-specific expansions and homologous gene loss/gain of *GH3* gene family during the evolutionary process. In addition, selective force and expression analyses indicated that all *GH3* genes were constrained by strong purifying selection, and *GH3* genes in conserved Ors showed higher expression levels than that in unconserved Ors. The current study highlighted different evolutionary patterns of *GH3* genes in Gramineae crops resulted from different evolutionary rates and duplication events and provided a vital insight into the functional divergence of *GH3* genes.

## Introduction

Auxin, one of the most important hormones in plants, plays essential roles in embryogenesis, vascular differentiation, phototropism, and plant morphology (Jain et al., 2006; Terol et al., 2006). *Glycoside hydrolase 3* (*GH3*) gene family belongs to auxin-responsive gene families and directly influences the homeostasis of different plant hormones (Vielba, 2018; Kong et al., 2019a). Previous studies demonstrated that GH3 proteins can catalyze various reactions using indole-3-acetic acid (IAA), jasmonic acid (JA), benzoates, and salicylic acid (SA) as substrates (Staswick et al., 2002; Okrent and Wildermuth, 2011; Kong et al., 2019a). In plants, the number of GH3 protein members varies greatly, namely, 19 in *Arabidopsis thaliana* (Okrent et al., 2009), 13 in *Oryza sativa* ssp. *japonica* (Jain et al., 2006; Terol et al., 2006; Fu et al., 2011; Kong et al., 2019a), 13 in *Zea mays* (Feng et al., 2015), 15 in *Solanum lycopersicum* (Kumar et al., 2012), two in *Physcomitrella patens* (Zhang et al., 2018), and 18 in *Selaginella moellendorffii* (Zhang et al., 2018).

At present, GH3 proteins have been divided into three groups based on evolutionary analysis, sequence similarity, and substrate specificities: group I with JA and/or SA–amido synthetase, group II with IAA–amido synthetase activity, and group III with unknown synthetase activity (Staswick et al., 2002; Okrent et al., 2009). Plant GH3 proteins played important roles in signaling pathways, organ developments, and plant architecture (Fu et al., 2011; Singh et al., 2015; Cano et al., 2018). Among them, *OsGH3-1*, *OsGH3-2*, *OsGH3-8*, and *OsGH3-13* are associated with crosstalk modulation between the IAA, JA, and SA signaling pathways under biotic or/and abiotic stresses (Ding et al., 2008; Domingo et al., 2009; Zhang et al., 2009). *OsGH3-2* participates in drought and cold tolerances *via* regulating the levels of auxin and abscisic acid (ABA) (Ding et al., 2008; Du et al., 2012). *OsGH3-8* was reportedly associated with rice floret fertility and disease resistance (Ding et al., 2008; Jian et al., 2010; Yadav et al., 2011). Another study reported that “auxin-*miR167-ARF8-OsGH3-2*” signaling pathway responses to exogenous auxin (Yang et al., 2006). Additionally, “*miR156f-OsSPL7-OsGH3-8*” signaling pathway modulates the plant architecture (Dai et al., 2018).

As we all know, Gramineae is a large and nearly ubiquitous family of monocotyledonous flowering plants known as grasses and contains many important cereal crops with high economic value, well-characterized phylogeny, and numerous genetic resources for genetic engineering. Gramineae therefore is an exceptional model system to study short-term evolutionary dynamics of gene families in the plants. In this study, six important Gramineae crops (*Brachypodium distachyon*, *Hordeum vulgare*, *Setaria italica*, *Sorghum bicolor*, *Z. mays*, and *O. sativa* ssp. *japonica*) were selected to study expansion and evolutionary patterns of *GH3* genes at the genome-wide scale and to systematically analyze *GH3* genes’ phylogenetic relationship, chromosomal location, duplication events, orthogroups (Ors), selective force, gene structure, protein motifs, *cis*-elements in promoters, and expression profiles. Our results could provide a valuable foundation for further investigation of gene expansion, evolutionary patterns, and functional differentiation in the Gramineae *GH3* gene family.

## Materials and Methods

### Identification and Phylogenetic Analysis of *GH3* Genes

Genome datasets of *B. distachyon* (v3.0), *H. vulgare* (IBSC_v2), *S. italica* (v2.0), *S. bicolor* (NCBIv3), *Z. mays* (B73_RefGen_v4), and *O. sativa* ssp. *japonica* (MSU 7.0) were downloaded from Ensembl Plants (http://plants.ensembl.org/index.html) and TIGR Database (http://rice.plantbiology.msu.edu), respectively. The HMM (Hidden Markox Model) profile of the GH3 auxin-responsive promoter (PF03321) was obtained from Pfam (http://pfam.xfam.org/). All GH3 proteins were separately searched by HMMER 3.2.1 (with default parameters) and BLASTP (E-value of e^−5^) methods (Kong et al., 2019a). Subsequently, all candidate sequences were subjected to SMART (http://smart.embl-heidelberg.de/) and Pfam (http://pfam.xfam.org/search/sequence) for key conserved domain checks (Singh et al., 2015; Deng et al., 2019), and the candidate GH3 protein sequences without the GH3 auxin-responsive promoter (PF03321) were filtered out (Kong et al., 2018).

All identified GH3 proteins were aligned by ClustalW, and a phylogeny tree was generated in MEGA 6.0 using the neighbor-joining method with 1,000 bootstrap replicates (Zhang et al., 2018). Names of putative *GH3* genes were assigned principally based on chromosomal order in each genome in accordance with previous rice *GH3* gene study (Jain et al., 2006; Terol et al., 2006; Kong et al., 2019a).

### Chromosomal Locations, Gene Structure, and Conserved Motifs

The data about chromosomal locations and gene structures of *GH3* genes in all tested species were obtained from GFF3 files and shown by TBtools v0.665 (Chen et al., 2018). Conserved motifs of all GH3 proteins were investigated by MEME Suite 5.0.2 (http://meme-suite.org/tools/meme) with the maximum number of motif sets at 20, the optional width of motifs from 6 to 100 amino acids, and other default parameters (Zhang et al., 2018; Kong et al., 2019b). Next, all identified motifs were annotated by Pfam and InterProScan (http://www.ebi.ac.uk/interpro/search/sequence-search) databases (Mitchell et al., 2018).

### Gene Duplication Events, Orthogroups Identification, and Selective Forces

Gene duplication events of the *GH3* gene family were analyzed and classified by the “duplicate_gene_classifier” script in MCScanX with an E-value of 1e^−5^ in BlastP search (Kong et al., 2019b). In general, whole genome duplication (WGD) or segmental duplication gene pairs were defined when the pairs of genes within two segmental regions have collinearity. When two duplicate genes were consecutive, we considered the gene pairs as tandem duplications. When two duplicate genes were separated by a maximum of 20 gene loci, we considered the gene pairs as proximal duplications. Within two duplicate genes from one duplication event, the synonymous (Ks)/nonsynonymous (Ka) substitution (Ka/Ks) rate was analyzed by DnaSP 5.0 (http://www.ub.edu/dnasp/) (Librado and Rozas, 2009), and divergence time was estimated by *T*  =  Ks/(2 × 9.1 × 10^−9^) × 10^−6^ Mya program (Deng et al., 2019; Kong et al., 2019a).

In this study, OrthoFinder software was used to identify *GH3* Ors (Emms and Kelly, 2015). An all-vs-all BlastP search was conducted by diamond software (Buchfink et al. 2015) (https://ab.inf.uni-tuebingen.de/software/) with the following parameters: E-value of 1e^−3^ as the input file for OrthoFinder software, and Ors then were identified according to the published methods (Emms and Kelly, 2015). Then, Tajima’s *D* values of all Ors were calculated by DnaSP 5.0 for selective forces analysis among Ors (Librado and Rozas, 2009).

### *cis*-Elements and Expression Analyses of *GH3* Genes

Two-Kbp upstream promoter sequences from the start codon (ATG) of all *GH3* genes in these six tested species were submitted to the PLANTCARE website (http://bioinformatics.psb.ugent.be/webtools/plantcare/html) for the analysis of *cis*-acting regulatory elements (*cis*-elements) (Lescot et al., 2002; Liu et al., 2016).

Raw datasets of rice seven essential tissues (DRX000661, DRX000663, DRX000665, DRX000667, DRX000669, DRX000671, and DRX000673) were downloaded from NCBI (https://www.ncbi.nlm.nih.gov/) and analyzed according to previously published article (Kong et al., 2019a). The fragment per kilobase of exon per million fragments mapped (FPKM) method was adopted to calculate gene expression levels (Kong et al., 2018). In addition, expression date of maize 79 tissues at different development stages was obtained from maize eFP Browser (Hoopes et al., 2018) (data source: Hoopes et al. Atlas, http://bar.utoronto.ca/efp_maize/cgi-bin/efpWeb.cgi). In order to detect the response of *GH3* genes to biotic and abiotic stresses, the expression data of maize *GH3* genes under salt, cold, heat, and *Colletotrichum graminicola* was downloaded from maize eFP Browser (Hoopes et al., 2018) (Data Source: Hoopes et al. Stress).

## Results

### Identification and Classification of *GH3* Genes in Gramineae Crops

**Figure 1:**
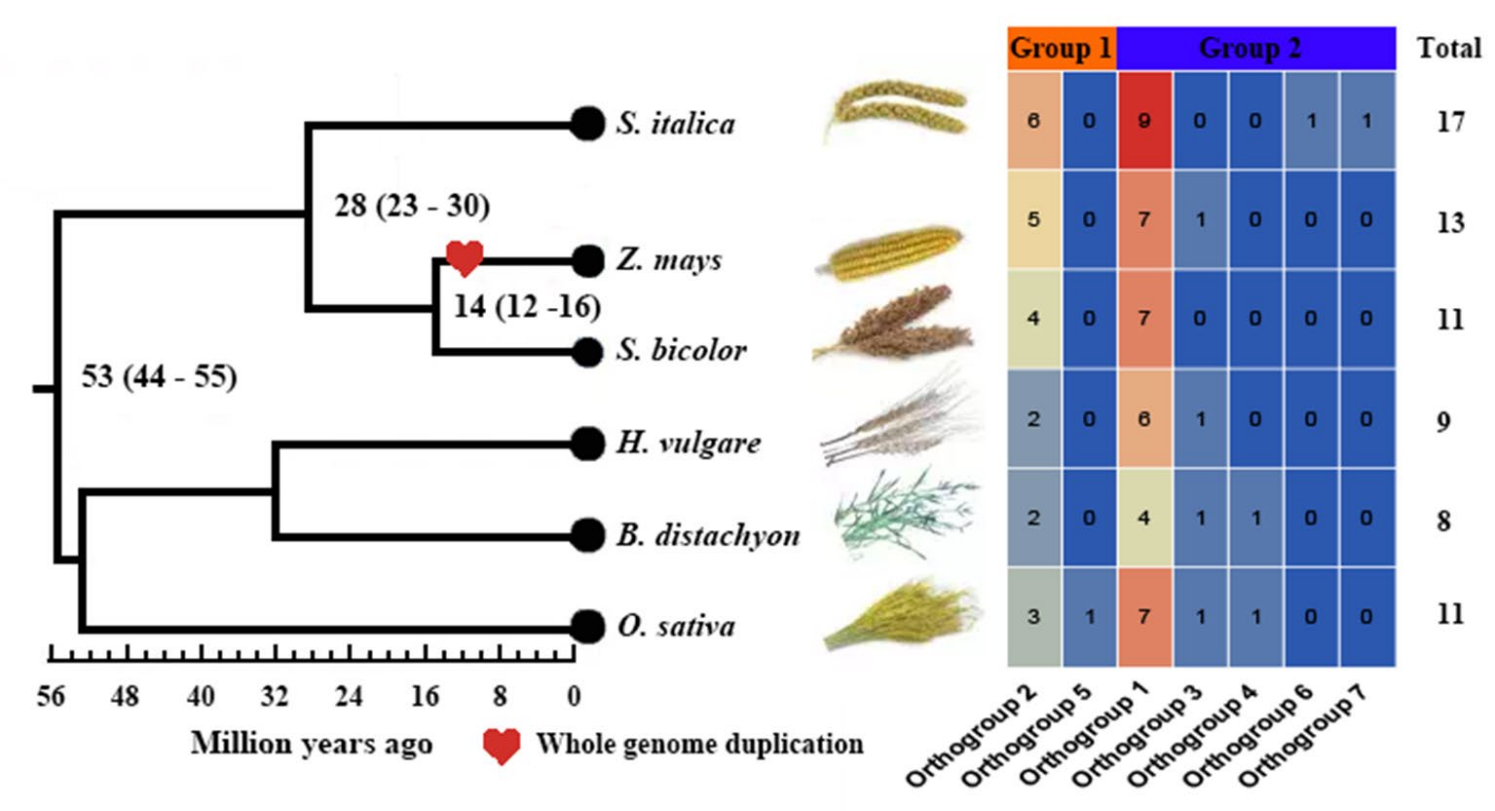
Comparison of the number of *GH3* genes in six Gramineae crops. Groups I and II are displayed in different colored boxes. The numbers of orthogroups are shown by heat map: blue means low, and red means high.

**Figure 2:**
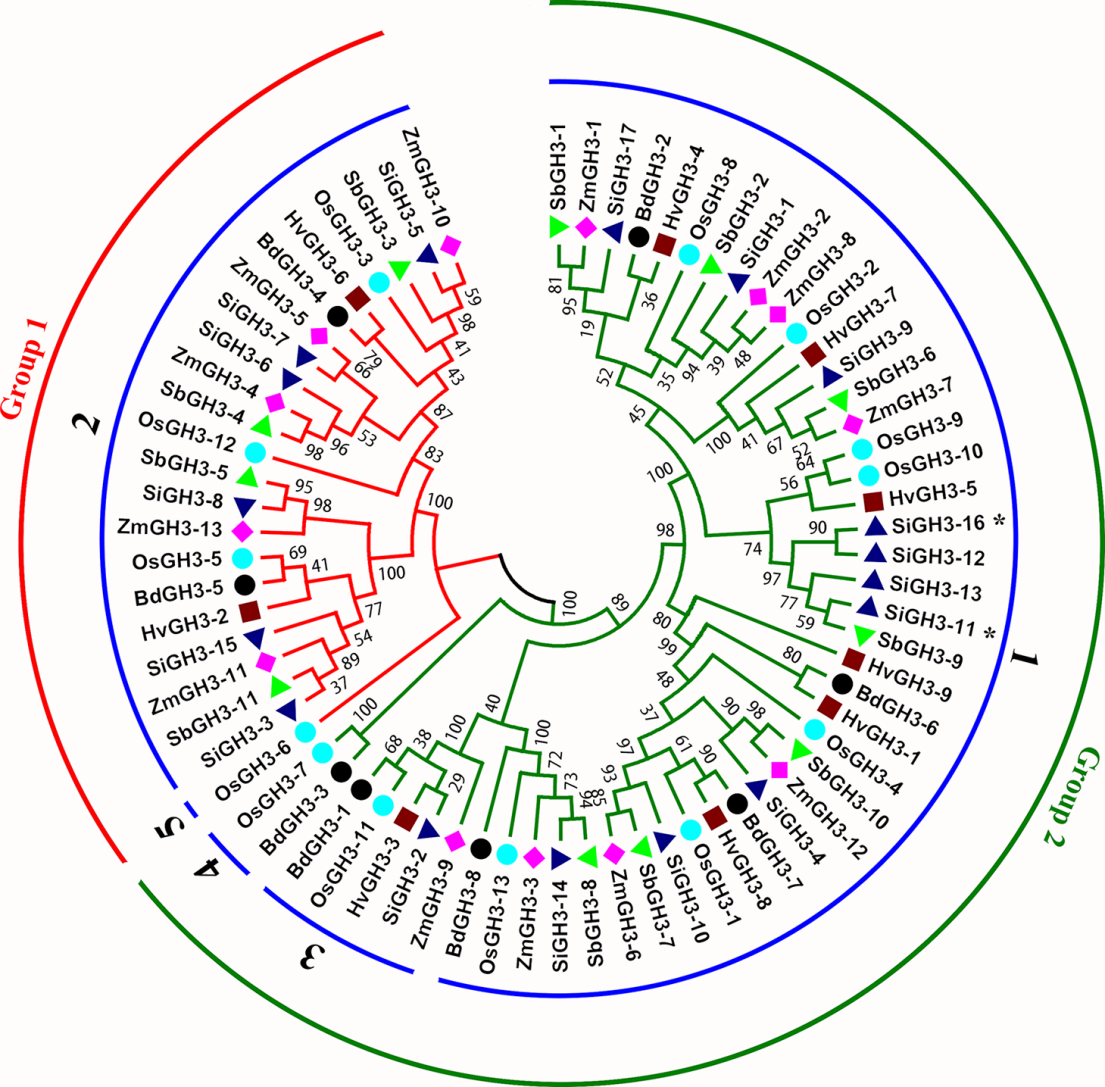
A phylogeny tree of GH3 protein sequences from *H. vulgare*, *B. distachyon*, *O. sativa* ssp. *japonica*, *S. italica*, *S. bicolor*, and *Z. mays*. Different colors of circles represent different groups. The numbers on blue circles represent different orthogroups, such as 1 means Orthogroup1. The different species are displayed by different shaped markers. SiGH3-11 * and SiGH3-16 belonged to Orthogroup6 and Orthogroup7, respectively.

We investigated a total of 69 nonredundant *GH3* genes from the six studied species, with 17 in *S. italica*, 13 in *Z. mays*, 11 in *S. bicolor*, 9 in *H. vulgare*, 8 in *B. distachyon*, and 11 in *O. sativa* ssp. *japonica* (**Table S1**). *S. italica* had more *GH3* genes than other tested species, namely, *H. vulgare* and *B. distachyon* (**Figure 1**, **Table S1**). To reveal the number difference of *GH3* genes in these species, group classification and Or identification were conducted. Results showed that all *GH3* genes grouped into two groups (I and II) consisted of seven Ors (**Figures 1** and **2**, **Table S2**). Further studies indicated that the number of Ors varies greatly among these six species. Or1 and Or2 were existed in all tested species, while Or5, Or3, Or4, Or6, and Or7 were lineage-specific, whereas Or5 was only in *O. sativa* ssp. *japonica*, and Or6 and Or7 were only in *S. italica* (**Figure 1**). The Or1 and Or2 have more genes than other Ors, and these two Ors existed in all tested crops. We thus defined these two Ors as conserved Ors, whereas others as unconserved Ors. What’s more, we further noticed that gene numbers of these two Ors were different between different crops. For example, Or1 showed four to six genes in *S. italica*, *Z. mays*, and *S. bicolor*, whereas only two to three genes in *H. vulgare*, *B. distachyon*, and *O. sativa* ssp. *japonica*. These results indicated that the *GH3* gene family has lineage-specific expansions and homologous gene loss/gain in Gramineae crops during the evolutionary process.

### Chromosomal Locations and Gene Duplication Events

**Figure 3:**
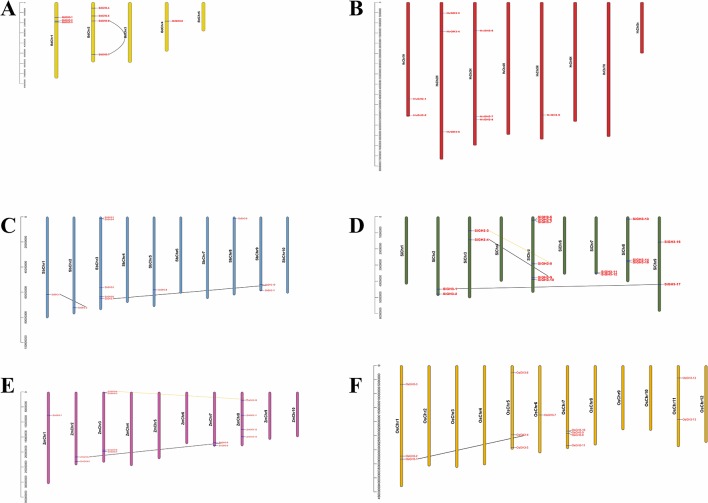
The chromosome location and duplication events of *GH3* genes in six species, namely, *B. distachyon* **(A)**, *H. vulgare* **(B)**, *S. bicolor* **(C)**, *S. italica* **(D)**, *Z. mays* **(E)**, and *O. sativa* ssp. *japonica* **(F)**. The yellow and black lines represent WGD or segmental duplication events in Orthogroup2 (Or2) and Orthogroup1 (Or1), respectively.

To understand the expansion mechanism of paralogues, we investigated gene locations and duplication modes within each species. Of all tested species, *GH3* genes were unevenly distributed on all chromosomes (Chrs) (**Figure 3**). For example, there were three, four, and one *GH3* gene in Chr1, Chr2, and Chr4, respectively, while no *GH3* gene in Chr3 and Chr5 (**Figure 3A**). Interestingly, barley *GH3* genes are localized at the proximal regions of the chromosomes, which are the dynamic parts of the chromosomes and very prone to segmental or tandem duplications (Ablazov and Tombuloglu, 2016; Bostancioglu et al., 2018; Tombuloglu, 2018; Tombuloglu et al., 2019). Additionally, WGD or segmental duplication events were found in *B. distachyon*, *S. bicolor*, *S. italica*, *Z. mays*, and *O. sativa* ssp. *japonica*, while tandem duplication, proximal, and dispersed duplication events were not found among these crops (**Figures 3A–F**). This result suggested WGD or segmental duplication events were the main gene expansion mode of *GH3* gene family in Gramineae crops. Interestingly, WGD or segmental duplication events existed only in Or1 (seven pairs) and Or2 (two pairs) (**Figure 3**), indicating that gene expansions had Or specificity.

Analysis of the Ka/Ks rates of duplicate gene pairs (**Table S3**) revealed that Ka/Ks ratios of all duplicate gene pairs had less than 1, suggesting that they underwent purifying selection according to the neutral theory. Divergence times of all duplicate gene pairs ranged from 5.43 to 27.70 Mya, except for *SiGH3-3* and *SiGH3-8* (61.43 Mya) (**Table S3**). These results indicated that duplication events happen in different stages and play essential roles of *GH3* gene family expansions.

### Sequence Characteristics and Intron Number Analyses

**Figure 4:**
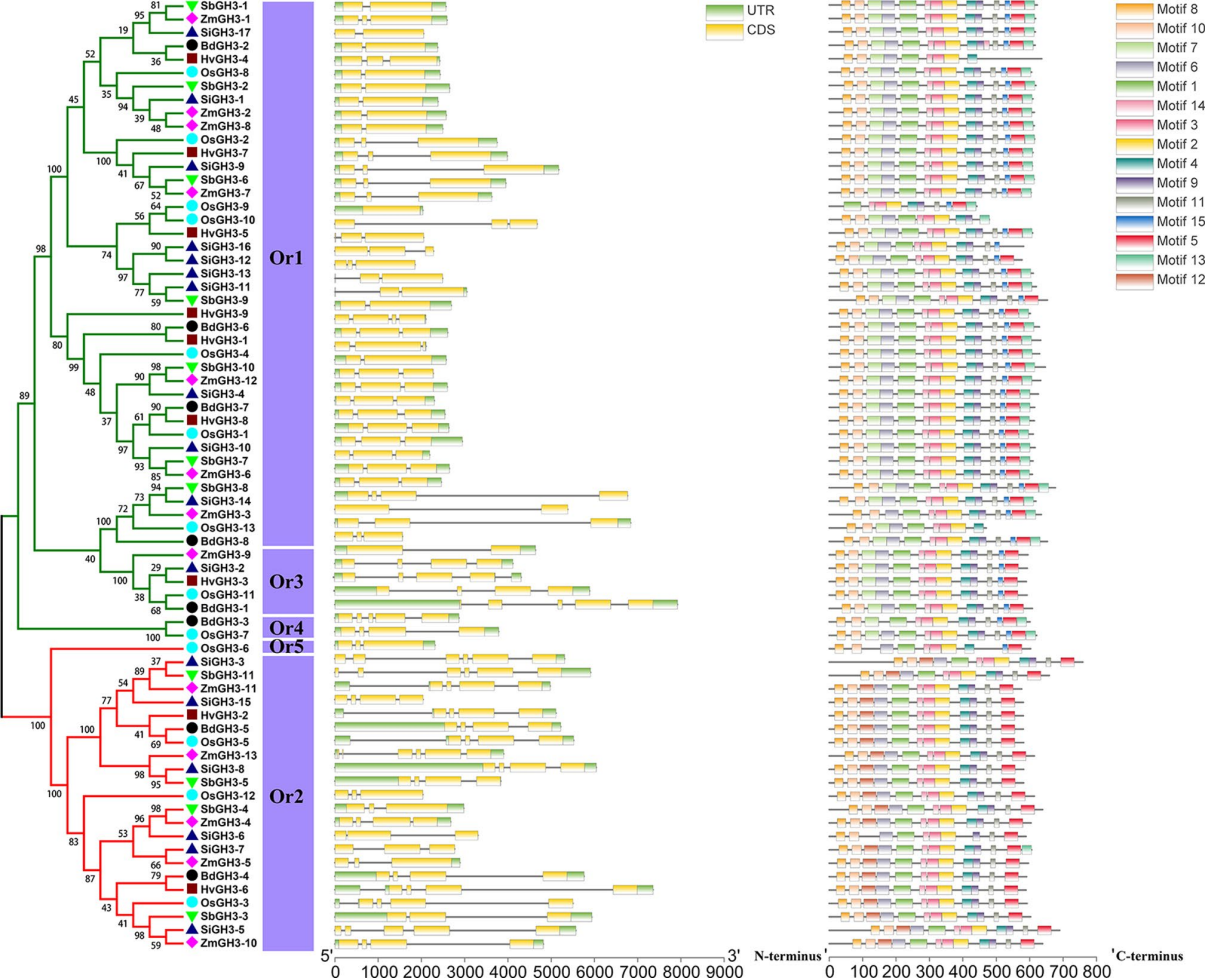
The phylogenetic tree, exon/intron structure, and conserved motif compositions of the *GH3* genes in these six species. A phylogeny tree of GH3 protein sequences from *H. vulgare*, *B. distachyon*, *O. sativa* ssp. *japonica*, *S. italica*, *S. bicolor*, and *Z. mays*. Different colors of branches represent different groups. The different species are indicated by different shaped markers, and Or means orthogroup. The relative lengths of genes and proteins are shown by the widths of the gray bars. The exons and introns are displayed by yellow boxes and gray lines, respectively.

In this study, 20 conserved motifs (**Figure 4**, **Table S4**) were found using MEME suite. We observed that all GH3 proteins showed the similar motifs arrangement, and most identified conserved motifs were parts of GH3 auxin-responsive promoter domain (**Table S4**), implying that protein sequences of *GH3* gene family members are very conservative among different groups/Ors in these tested Gramineae crops. Compared to motifs arrangement, intron/exon structures of all *GH3* genes were obviously different between different groups, even in Ors. Phylogenetic tree, exon/intron numbers in CDS, and identified Ors could divide all *GH3* genes into obvious classification. Genes in Or2, Or3, and Or4 contained more exons in CDS than Or1 and Or5. These results revealed that *GH3* genes from different Ors have been differentiated.

### Selective Forces Analysis, Expression, and Function Divergences

To elucidate the evolutionary forces and diverged patterns of different *GH3* Ors among Gramineae crops, Tajima’s *D* and average expression values of Ors were calculated. We found all Ors were under strong purifying selection and ranged from −0.3 to −0.9049 (**Table S5**). These results pointed out that genes in different Ors faced different evolutionary rates and purifying selection pressures during the evolutionary process.

**Figure 5:**
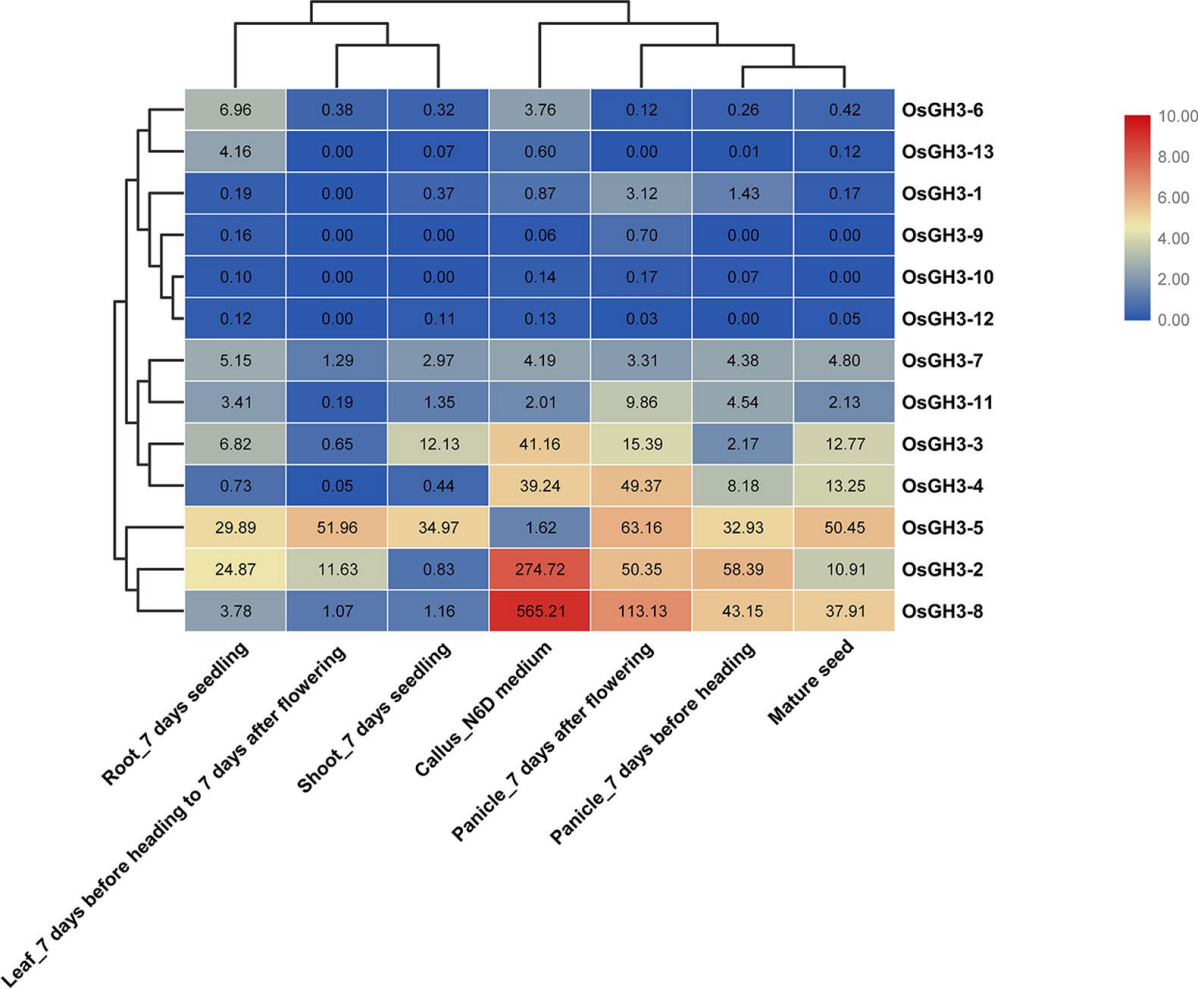
Expression profiles of *OsGH3* genes in different tissues. The values in the color scale represent log_2_^FPKM^: red/green indicates high level/low level of transcript abundance.

Expression patterns of seven essential tissues showed that conserved Ors (Or1 and Or2) had higher average expression values than unconserved Ors (Or3, Or4, and Or5) (**Table S5**) in rice and maize. Further studies showed that *OsGH3-2*, *OsGH3-4*, and *OsGH3-8* (all in Or1) had similar expression with high expression levels in callus, panicle, and mature seed, while *OsGH3-3* and *OsGH3-5* (both in Or2) showed completely different expression profiles (**Figure 5**). This result indicated that genes in Or1 may be redundant, whereas genes in Or2 have functional differentiation. Interestingly, we detected the expression patterns of one WGD or segmental duplication event (*OsGH3-1* and *OsGH3-4*). *OsGH3-1* was not expressed in any tissue (**Figure 5**), indicating this gene had undergone pseudogenization after the duplication event. Differently, in maize, *ZmGH3-2* and *ZmGH3-8* (one WGD or segmental duplication event) showed similar expression patterns (**Figure S1** and **Table S6**), implying that these two genes are functionally redundant. In addition, we found that the remaining maize *GH3* genes showed obviously different expression patterns. For example, *ZmGH3-10* showed a high expression level in leaf; *ZmGH3-12* was highly expressed at meiotic tassel V18; *ZmGH3-7* was strongly expressed at anthers R1.

**Figure 6:**
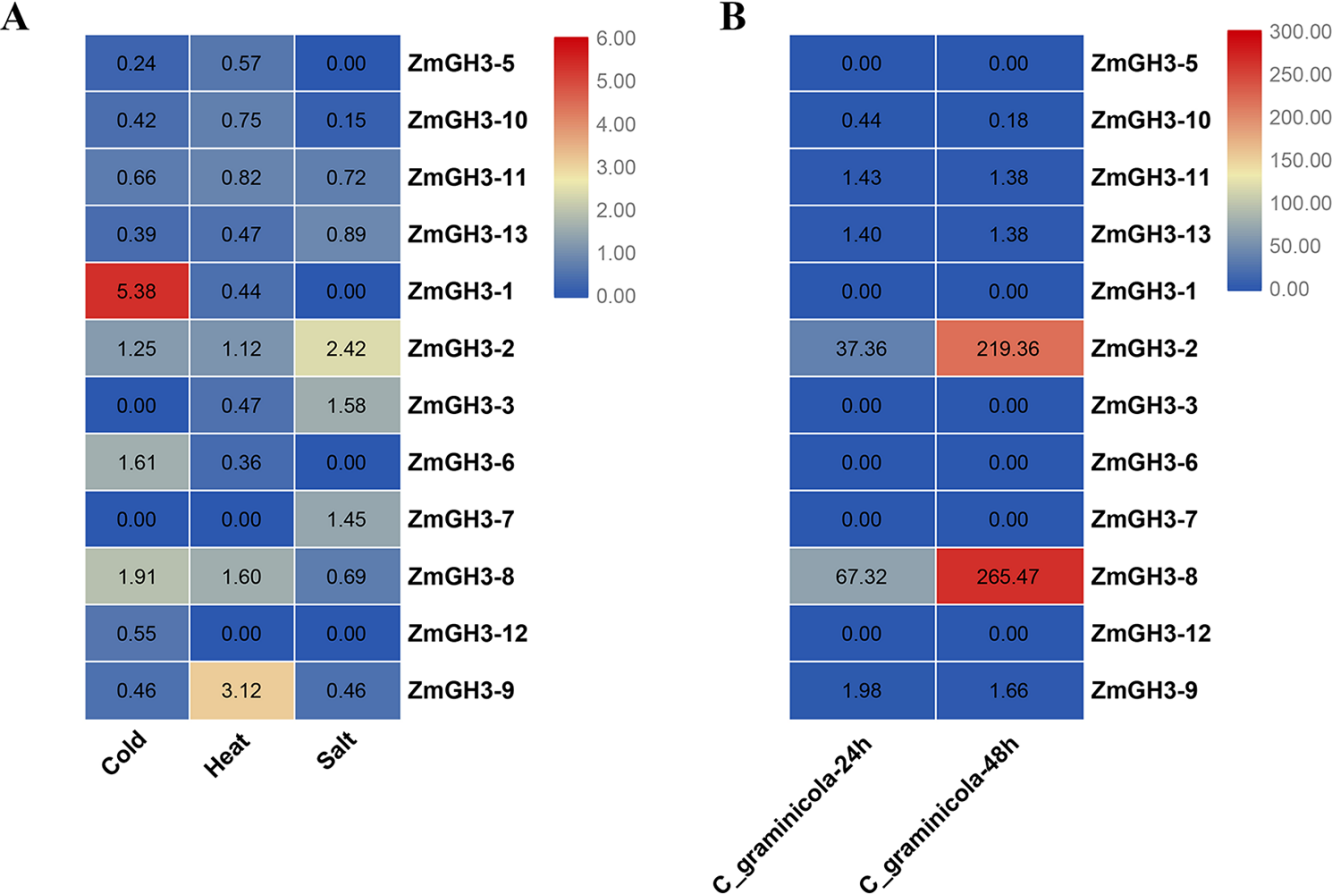
Expression change folds of *GH3* genes under salt, cold, and heat **(A)**, as well as after *C. graminicola* infection **(B)**. Expression change fold  =  expression value of treated sample/expression value of control sample. The values in the color scale represent change folds from lower (blue color) to higher (red color).

Previous studies reported that *GH3* genes play important roles in biotic and abiotic stresses (Fu et al., 2011; Zhang et al., 2018; Kong et al., 2019a). We thus examined the expression changes of maize *GH3* gene under salt stress, cold stress, heat stress, and after *C. graminicola* infection (**Figure 6** and **Table S7**). Under salt, cold, and heat stresses, most maize *GH3* genes showed obvious down-regulation. However, *ZmGH3-1* showed high up-regulation under cold stress; *ZmGH3-9* was highly up-regulated under heat stress; *ZmGH3-2* showed clear up-regulation under salt stress; *ZmGH3-8* showed slight up-regulation under cold and heat stresses. Similarly, most maize *GH3* genes were also down-regulated after *C. graminicola* infection. Only two maize *GH3* genes (*ZmGH3-2* and *ZmGH3-8*) have quite high up-regulation (>30).

**Figure 7:**
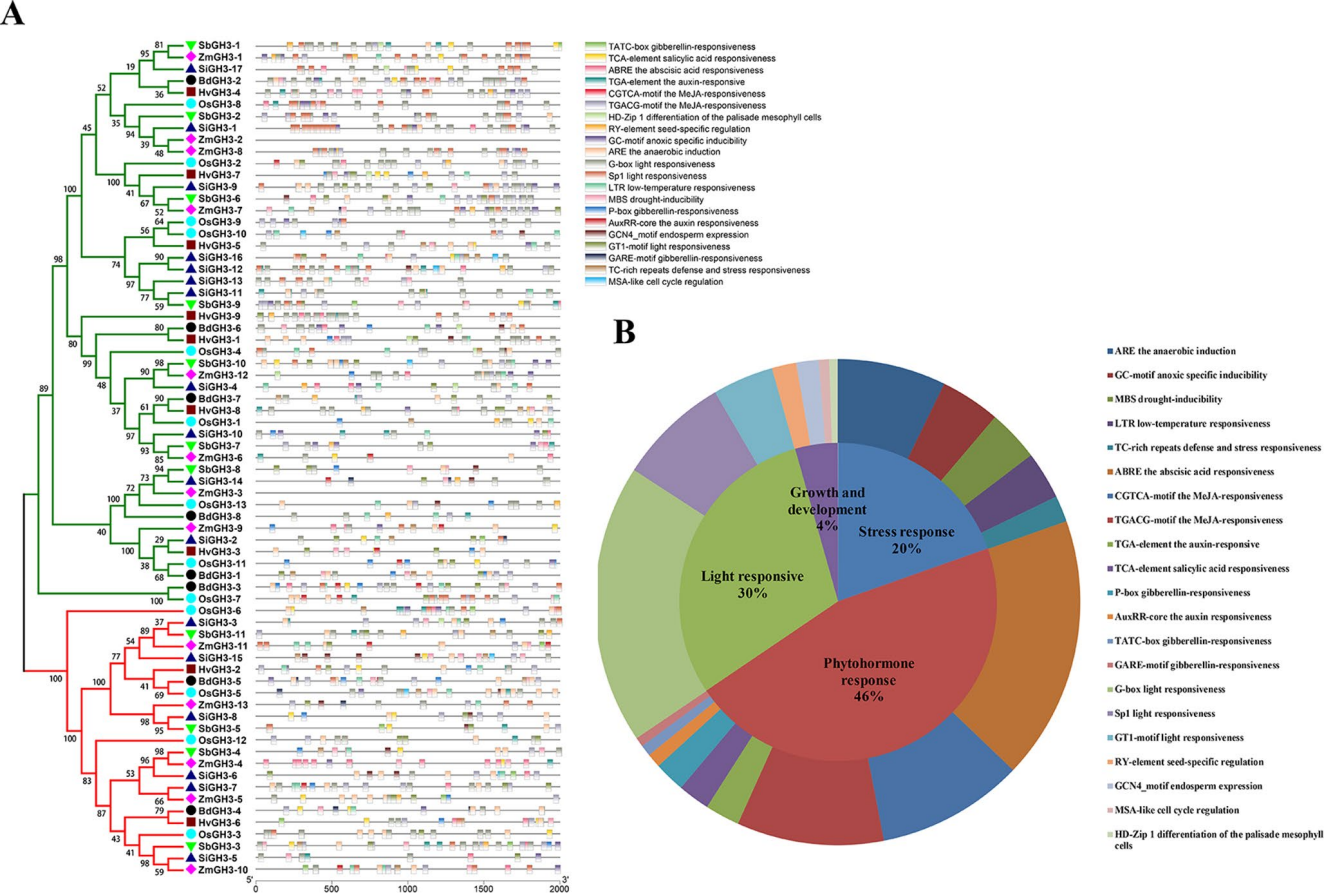
*cis*-Acting elements in all *GH3* genes in Gramineae crops. **(A)** *cis*-Acting elements of all *GH3* genes showed according to a phylogenetic tree. The different colored boxes indicate different promoter elements in each *GH3* gene. **(B)** Pie charts of different sizes represent the ratio of each promoter element in each category.

The *cis*-elements in the gene promoter regions can regulate gene expression, which provides an important insight into gene function prediction. A total of 21 *cis*-elements were identified through the PLANTCARE website (Lescot et al., 2002) and grouped into four categories, namely, light responsive, growth and development, stress responsive, and phytohormone response (**Figure 7**). The positioning result of *cis*-elements showed that *cis*-elements were unevenly distributed on promoters of all *GH3* genes (**Figure 7A**). We found that 46% of elements belonged to phytohormone response category and were involved in auxin responsiveness (TGA-element and AuxRR element), ABA responsiveness (ABRE), MeJA responsiveness (CGTCA motif and TGACG motif), SA responsiveness (TCA element), and gibberellin responsiveness (P-box, TATC box, and GARE) motifs (**Figure 7B**). Twenty percent of elements belonged to stress response category associated with multiple stresses, such as drought (MBS) and low temperature (LTR) (**Figure 7B**). In addition, G-box, Sp1, and GT1 motif were in light responsive category and covered 30% of all elements (**Figure 7B**). Furthermore, four elements (RY-element, GCN4 motif, MAS-like, and HD-Zip1) in growth and development category related to several processes (**Figure 7B**). Considering these results, we proposed that *GH3* genes participate in light responsive, phytohormone response, stress response, and plant growth and development.

## Discussion

### Evolution of *GH3* Genes in Gramineae Crops

In the current study, 69 *GH3* genes from six Gramineae crops were identified. We found no direct relevance between genome sizes and the number of *GH3* genes. For example, there were 17 *GH3* genes in *S. italica* (genome size: 490 Mbp), while there were nine genes in *H. vulgare* (genome size: 4.79 Gbp). In addition, there was no direct relevance between WGDs and the number of *GH3* genes. Previous studies reported that *Z. mays* underwent one specific WGD more than other Gramineae plants (Swigoňová et al., 2004). However, *Z. mays* had fewer *GH3* genes than *S. italica* in this study. In addition, numbers and sizes of Ors vary greatly among these tested Gramineae crops, indicating that the *GH3* gene family has undergone different evolutionary approaches in different species, and this conclusion is also supported by our Or identification and selective force results. Our results supported theoretical models of gene family evolution that gene families continuously undergo stochastic gain and loss events (Zimmer et al., 1980; Reed and Hughes, 2004; Hahn et al., 2005). It is well known that gene tandem duplication, segmental duplication, transpositions, and WGDs have significant roles in biological evolution (Xu et al., 2012). In our study, we observed that duplicate gene pairs developed through WGD or segmental duplication, while no gene pair was observed from other mode duplications, specifying that WGD or segmental duplication has played an important role in the expansion of *GH3* gene family in some Gramineae species. Our earlier study of *GH3* gene family evolution in *Oryza* genus also verified this conclusion (Kong et al., 2019a). Moreover, we noticed that the divergence times of all duplicate gene pairs ranged from 5.43 to 61.43 Mya, including *OsGH3-1* and *OsGH3-4* (23.48 Mya). Our previous study in *Oryza* genus also showed the divergence time of *GH3-1* and *OsGH3-4* homologous gene pair, which ranged from 23.07 to 31.01 Mya (Kong et al., 2019a). These results indicated that WGD or segmental duplication events play a more important role in *B. distachyon*, *S. italica*, *Z. mays*, and *S. bicolor* than *H. vulgare* and *O. sativa* ssp. *japonica*.

### Functional Diversity in *GH3* Genes

The *GH3* genes regulate diverse biological processes (Cano et al., 2018) and govern many fundamental aspects of plant growth and development (Liu et al., 2016; Vielba, 2018; Zhang et al., 2018; Kong et al., 2019a). In this study, many elements of light responsive, growth and development, stress responsive, and phytohormone response were identified and supported that *GH3* genes have the potential to regulate diverse plant growth and development processes. Selective forces (Tajima’s *D*) and public data gene expression analysis might help us to be informed on the functional diversity of *GH3* genes. We found different Ors were under differential selective forces values, which may lead to functional differentiation between these Ors. As expected, different Ors showed different expression patterns: conserved Ors showed higher expression levels and broader expression tissues than unconserved Ors. These expression patterns confirmed the functional differentiation of different Ors. In addition, conserved Ors showed obvious quantity expansion than unconserved Ors, such as Or2 versus Or5, and Or1 versus Or3. We thus speculate that expansions of Ors are related to plant adaptation to the environment. Current hypotheses proposed that duplicate gene pairs face four fates accompanied by function change after gene duplication events (Lynch and Force, 2000; He and Zhang, 2005; Conant and Wolfe, 2008): loss functions (pseudogenization), new functions (neofunctionalization), partitioning of the original functions (subfunctionalization), and subfunctionalization followed by neofunctionalization (subneofunctionalization). We found gene redundancy (genes in Or1), subfunctionalization (genes in Or2), and pseudogenization (*OsGH3-1* and *OsGH3-4*) in *GH3* gene family and supported functional diversity in *GH3* genes.

### *GH3* Genes Play Essential Roles in Plant Growth, Biotic, and Abiotic Stress Responses

Salt, cold, and heat are the major abiotic stresses that frequently affect the growth and development of plants under various natural conditions (Feng et al., 2015; Kong et al., 2019a; Kong et al., 2019b). Auxin-related transcriptional regulation is important for plants to survive and adapt to adverse environmental challenges (Feng et al., 2015; Kong et al., 2019a). For example, *OsGH3-2* can enhance drought and cold tolerances *via* regulating the levels of ABA (Ding et al., 2008; Du et al., 2012). Our earlier study also showed that *OsGH3-2* and *OsGH3-8* showed obvious up-regulation under salt stress and play important roles in rice salt tolerance (Kong et al., 2019a). In this study, our finding showed that *ZmGH3-1*, *ZmGH3-9*, *ZmGH3-2*, and *ZmGH3-8* responded to cold, heat, salt, and cold and salt stresses. These results evidenced that *GH3* genes are tightly associated with various abiotic stresses. In addition, our expression result also showed that *ZmGH3-2* and *ZmGH3-8* showed high up-regulation after *C. graminicola* infection (>30 fold), indicating these two genes play essential roles in *C. graminicola* response and tolerance. Overall, *ZmGH3-2* and *ZmGH3-8* may be good candidate genes for genetic engineering breeding against various biotic and abiotic stresses.

## Conclusions

In summary, diverged patterns of *GH3* genes in six Gramineae crops were observed from many aspects, including phylogenetic relationship, chromosomal location, duplication events, Ors, selective force, gene structure, protein motifs, *cis*-elements, and expression patterns. These results revealed that lineage-specific expansions and homologous gene loss/gain occurred during the evolutionary process. *GH3* gene family in Gramineae crops was constrained by strong purifying selection. Different selective values of Ors might be related to functional diversity. Taken together, our results will interpret a comprehensive understanding of the evolution of the *GH3* gene family in Gramineae crops.

## Data Availability Statement

All datasets for this study are included in the manuscript/**Supplementary files**.

## Author Contributions

WK designed and performed the experiments. YZ, XD, SL, and CZ analyzed parts of the data or prepared parts of the figures and tables. YL provided guidance on the whole manuscript. All authors reviewed and approved the final submission.

## Funding

This study was funded by the National Key Research and Development Program of China (grant 2016YFD0100400) and the National Special Key Project for Transgenic Breeding (grant 2016ZX08001001).

## Conflict of Interest

The authors declare that the research was conducted in the absence of any commercial or financial relationships that could be construed as a potential conflict of interest.

## Supplementary Material

Table S1 Summary of information on GH3 genes in six Gramineae crops.

Table S2 Orthogroups among in six Gramineae crops.

Table S3 Ka/Ks ratios and divergence times of duplicate gene pairs.

Table S4 Annotation of conserved motifs in all GH3 proteins.

Table S5 Tajima’s D and average expression values of Ors. **(A)** Tajima’s D of each Or was calculated by DnaSP 5.0 (Librado and Rozas, 2009). **(B)** Average expression values (average FPKM value = total FPKM value of all genes in Or/gene number in Or) of all rice Ors. **(C)** Average expression values (the same calculation method to Figure B) of all maize Ors.

Table S6 The expression values of maize *GH3* genes in 79 tissues at different stages.

Table S7 The expression values of maize *GH3* genes under salt, cold, and heat, as well as after *C. graminicola* infection.

Figure S1 Expression profiles of *ZmGH3* genes in various tissues at different stages. The values in the color scale represent expression values: red/green indicates high level/low level of transcript abundance.
