# Supplementary figures and images for: Comparative Genomic and Transcriptomic Analysis Suggests the Evolutionary Dynamic of GH3 Genes in Gramineae Crops
Source: Front Plant Sci. 2019 Oct 15;10:1297. doi: 10.3389/fpls.2019.01297 (PMC6803601; doi:10.3389/fpls.2019.01297)

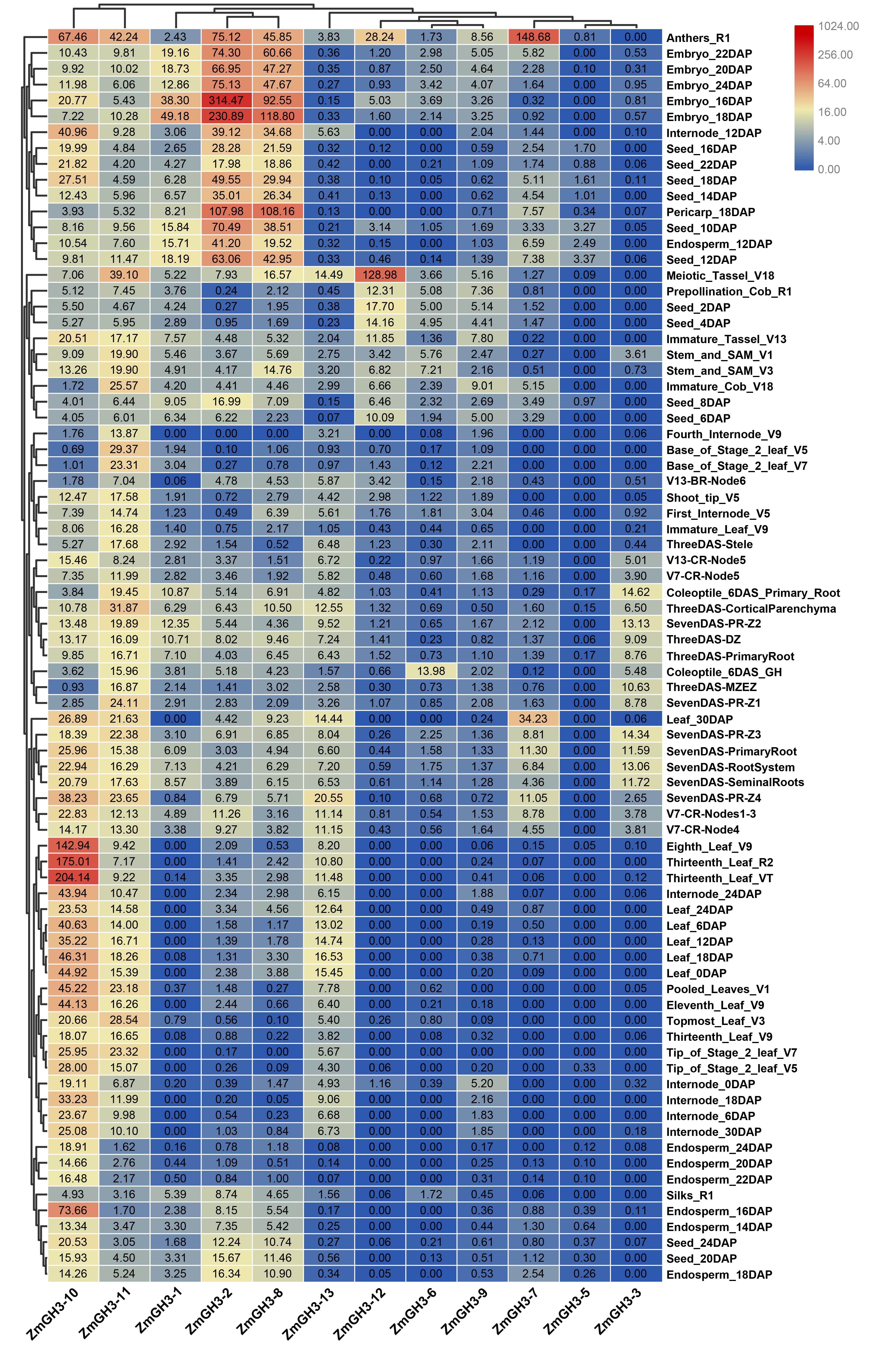

Supplement: Figure S1 — Expression profiles of ZmGH3 genes in various tissues at different stages. The values in the color scale represent expression values: red/green indicates high level/low level of transcript abundance. [file Image_1.jpeg]
